# Wireless Power Transfer for Autonomous Wearable Neurotransmitter Sensors

**DOI:** 10.3390/s150924553

**Published:** 2015-09-23

**Authors:** Cuong M. Nguyen, Pavan Kumar Kota, Minh Q. Nguyen, Souvik Dubey, Smitha Rao, Jeffrey Mays, J.-C. Chiao

**Affiliations:** Electrical Engineering Department, University of Texas at Arlington, TX 76019, USA; E-Mails: pavankumar.kota@mavs.uta.edu (P.K.K.); minh.nguyen66@mavs.uta.edu (M.Q.N.); souvik.dubey@mavs.uta.edu (S.D.); smitha@uta.edu (S.R.); jeffrey.mays@mavs.uta.edu (J.M.); jcchiao@uta.edu (J.-C.C.)

**Keywords:** wireless power transmission, multi-transmitter antenna, neurotransmitter sensor recorder, L-glutamate sensors

## Abstract

In this paper, we report a power management system for autonomous and real-time monitoring of the neurotransmitter L-glutamate (L-Glu). A low-power, low-noise, and high-gain recording module was designed to acquire signal from an implantable flexible L-Glu sensor fabricated by micro-electro-mechanical system (MEMS)-based processes. The wearable recording module was wirelessly powered through inductive coupling transmitter antennas. Lateral and angular misalignments of the receiver antennas were resolved by using a multi-transmitter antenna configuration. The effective coverage, over which the recording module functioned properly, was improved with the use of in-phase transmitter antennas. Experimental results showed that the recording system was capable of operating continuously at distances of 4 cm, 7 cm and 10 cm. The wireless power management system reduced the weight of the recording module, eliminated human intervention and enabled animal experimentation for extended durations.

## 1. Introduction

L-glutamate (L-Glu) is a widespread excitatory neurotransmitter in the central nervous system (CNS) and has received attention in medical research [1,2,3,4,5,6,7]. It has been implicated in fundamental functions of the sensory and motor systems including development [8,9], neuronal plasticity [10], learning [11,12], and memory [13,14,15,16,17]. *In vivo* measurement and monitoring of L-Glu in small animals could benefit studies of many acute and chronic neurological disorders that involve plastic changes in synaptic transmission [18,19] and excitotoxic neurological cell death [4,9,20]. Using implantable amperometric sensors has become a common method of real time L-Glu monitoring [4,21,22]. However, electrical currents from L-Glu are on the order of pico-amperes and require a recording system utilizing multiple high-gain amplifier stages. In addition, the recording system needs to be wearable for animals to minimize human intervention during experimentation [9,23,24]. The data from such front-end recording systems were wirelessly transmitted to the base station. A radio-frequency (RF) transceiver was thus implemented adding significant power consumption to the wearable recording system [23,25,26]. Therefore, there are remaining challenges in power consumption optimization and deployment of power supply methods [23].

Energy harvesting methods for wearable devices have emerged as an attractive solution for the aforementioned challenges, and can be categorized into either passive or active approaches. Passive approaches including harvesting energy from motion and vibration [27,28,29], thermal energy [30], photovoltaic energy [31] and ambient RF energy [32] allow energy collection from the surrounding environment. Although the sources are often available, harvested power is in the micro-watt range, which is insufficient to operate or activate a radio-frequency wireless transceiver module in wearable devices. Additionally, the passive sources are less reliable because they depend on the surrounding environment and offer a low conversion rate. For these reasons, they are not suitable as a lone source of energy [33]. On the other hand, active energy sources involve one or more external power sources such as batteries, super-capacitors and wireless power transmission (WPT) to supply power to wearable devices [24,27,28]. One of the main advantages of the active energy sources is the reliability since the sources are capable of maintaining normal device operation in long-term experiments. Using batteries for energy storage is a conventional approach; however, the operating life of the wearable device depends on battery capacity. In addition, the battery is not a preferred option in some cases as it increases the weight of the wearable module and is thus uncomfortable for the animal. WPT has recently emerged as one favorable alternative of supplying power to batteryless wearable devices. WPT could eliminate frequent battery replacements, minimize total weight and size of the wearable device, and support long term studies. Moreover, WPT could be conveniently optimized by antenna reconfiguration to satisfy power supply requirements.

WPT has been extensively studied for implantable devices such as pacemakers, cardiac implants [34,35], cochlear implants [36], esophageal pH monitoring for gastro-esophageal reflux disease (GERD) diagnosis [37], gastrostimulators [38], visual prosthesis [39], and neuromuscular stimulators [40]. In these studies, WPT systems were based on inductive coupling to transfer energy safely and efficiently between two resonant antennas. The systems were constructed for dedicated applications in which the positions of both transmitter and receiver antennas were carefully aligned with small relative displacements. In some applications such as wireless pacemakers or gastrostimulators, the positions of both antennas were fixed to the body to achieve high efficiency [35,37].

In this paper, we implemented a WPT system designed for the *in vivo* L-Glu recording module worn by a freely-moving rat. The rat was kept in an acrylic box, but the relative distance between the transmitter antenna and the receiver antenna carried by the rat varied randomly. We studied the lateral and angular misalignments between the two antennas and demonstrated the use of a multi-transmitter antenna configuration to extend transmission range and enhance effective coverage. A power management module was designed to maximize energy harvested from the receiver antenna to enable a low-power, low-noise and high-gain neurotransmitter recording system. An experiment demonstrated data acquisition from a MEMS-based L-Glu sensor and successful relay to the base station.

## 2. Experimental Section

### 2.1. Materials and Apparatus

#### 2.1.1. L-Glu Sensor Fabrication

**Figure 1:**
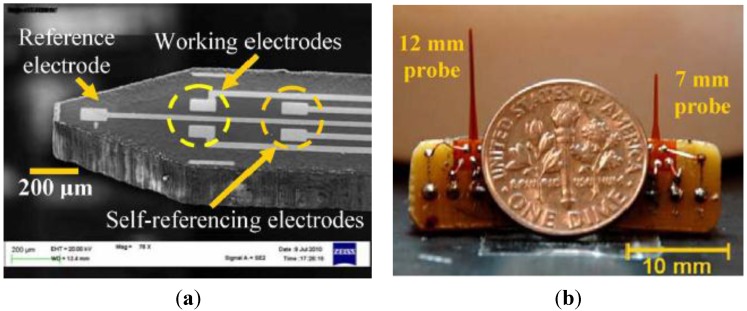
(**a**) Scanning electron microscopy (SEM) photo of the probe tip indicating two working electrodes (WE), two self-reference electrodes (SRE) and a reference electrode (RE) [4]; (**b**) A photo of the assembled devices with the probe lengths of 7 and 12 mm [4].

The implantable L-Glu sensors fabricated on a polyimide substrate with MEMS processes as described in our previous works [4,16] are briefly discussed here. The flexible polyimide substrate with a thickness of 125 µm was used to reduce tissue damage and scars caused by the implanted probes [4]. Standard MEMS-based processes were carried out to fabricate a multi-electrode array each with a sensing area of 50 × 100 µm^2^. After fabrication, each sensor probe was tailored using a laser machining process. The L-Glu sensor included a working electrode (WE), self-reference electrode (SRE) and reference electrode (RE). Figure 1a illustrates two L-Glu sensors on a probe with two separate WEs and SREs electrodes [4]. The RE was made of silver/silver chloride (Ag/AgCl) by chloridization of a silver electrode in a 1-M hydrochloric acid solution with saturated sodium chloride. The WE and SRE were made of gold using a physical deposition process [4]. The RE in this design was shared by the two L-Glu sensors to reduce probe dimensions [4]. Figure 1b shows a photograph of two probe designs with lengths of 7 and 12 mm. Copper wires were connected to each electrode of the probe using silver epoxy. The probe was connected to the neurotransmitter recording module, as described in the following section.

Since L-Glu is not an electroactive agent, the enzyme L-glutamate oxidase (GluOx) from USBio, Salem, MA, USA (G4001-01, 25-unit vial, 0.138 mg/unit) was coated on the WE [4,20]. In order to ensure the adhesion of the enzyme layer, cross-linking agent containing of 1% bovine serum albumin (Sigma-Aldrich, St. Louis, MO, USA) and 0.125% glutaraldehyde (Sigma-Aldrich, St. Louis, MO, USA) was mixed with the GluOx enzyme. The procedure of enzyme coating was described in our previous work [20]. Briefly, a micro-syringe (Hamilton Company, Reno, NV, USA) was utilized to carefully dispense the enzyme solution five times on the WE using a microscope. Afterwards, an organic layer of 1,3-phenylenediamine (mPD) (Sigma-Aldrich, St. Louis, MO, USA) was coated on the WE to form a selective membrane, which could improve the selectivity of the L-Glu sensor. It took 72 h for the protein matrix to cure completely on the electrode before use [4,20].

#### 2.1.2. Principle of Electrochemical L-Glu Sensors

The sensing mechanism of the L-Glu sensor follows the working principle of typical amperometric sensors in which the output current densities depend on the redox reaction rates occurring on their electrodes. In our sensors, L-Glu was turned into α-ketoglutarate and the byproduct hydrogen peroxide (H_2_O_2_) according to Equation (1) under the function of GluOx enzyme that was coated on the WE [4,20].

(1) $$\text{L}-\text{Glu}+{\text{ O}}_{2}+{\text{ H}}_{2}\text{O }\to \text{α}-\text{ketoglutarate}+{\text{NH}}_{3}+{\text{ H}}_{2}{\text{O}}_{2}$$

The amount of the electroactive byproduct H_2_O_2_ was detected by measuring current responses while maintaining a bias voltage of 0.7-V between the WE and RE. Hydrogen peroxide was oxidized in the following redox reaction:

(2) $${\text{H}}_{2}{\text{O}}_{2}\;\to {\text{ O}}_{2}+2{\text{H}}^{+}+2{\text{e}}^{-}$$

Meanwhile, a reduction reaction occurred on the RE:

(3) $$2\text{AgCl}+2{\text{e}}^{-}\;\to 2\text{Ag}+2{\text{Cl}}^{-}$$

Since the amount of Ag/AgCl on the RE was large and did not change during the experiments, the electrical current response only depended on the amount of hydrogen peroxide generated on the WE [41,42,43] and could be described as:

(4) $$\text{i}=\text{FA}\left\{\left[{\text{k}}_{{\text{H}}_{2}{\text{O}}_{2}}{\text{C}}_{{\text{H}}_{2}{\text{O}}_{2}}\left(0,\text{t}\right)\right]-\left[{\text{k}}_{{\text{O}}_{2}}{\text{C}}_{{\text{O}}_{2}}\left(0,\text{t}\right)\right]\;\left[{\text{k}}_{{\text{H}}^{+}}{\text{C}}_{H^{+}}(0,\text{t})\right]\right\}$$

where i denotes redox current generated by the L-Glu sensor, F is Faradays constant (96,485 C·mol^−1^), A is the area of the WE electrode, C_x_(0,t) is the surface concentration of chemical x, and k_x_ is the transport coefficient of reactant or product from the surface of the WE. According to Equation (4), the redox current i is proportional to the concentration of the reactant H_2_O_2_ generated from Equation (1). Therefore, monitoring the current output of such amperometric sensors could provide information of the L-Glu concentration on the surface of the WE.

In order to eliminate noise and interference from other electroactive analytes, the three-electrode configuration was utilized with a SRE. The SRE was designed and fabricated similar to the WE but was not coated with GluOx enzyme. Another amperometric sensor was established when a 0.7-V bias voltage was applied between the SRE and RE. This sensor recorded background noise and interference. Therefore, subtracting signals from the WE and SRE could provide a higher resolution in L-Glu measurements with higher sensor selectivity. This sensing method, known as the self-referencing technique [20,37,44], was incorporated in the hardware design of the neurotransmitter sensor module.

### 2.2. Miniature Wireless Recording System

The neurotransmitter recording system connected to the L-Glu sensor included sensor driven circuitry, signal processing modules and an RF transceiver. The sensor driven circuitry is described in [23]. Briefly, a constant and stable voltage of 0.7 V was generated using buffer circuitry. The RE had a potential of −0.7 V compared to the ground level of the WE and SRE. Electrical currents of the WE and SRE were amplified and converted into voltage signals using transimpedance amplifiers. The signal difference between the WE and SRE was further amplified through a low-noise, high-gain instrumentation amplifier. A system-on-chip (SoC) processor digitized voltage signals of the cascade amplifier system and transmitted these values to the base station. The recording system could operate with a supply voltage in a range of 1.6–3.0 V and current consumption of 17 mA. Equivalent input impedance was estimated to be 745 Ω in sleep mode. The recording system was designed to have a conversion gain of 10^9^ V/A that provided a sensing resolution of 10 pA.

**Figure 2:**
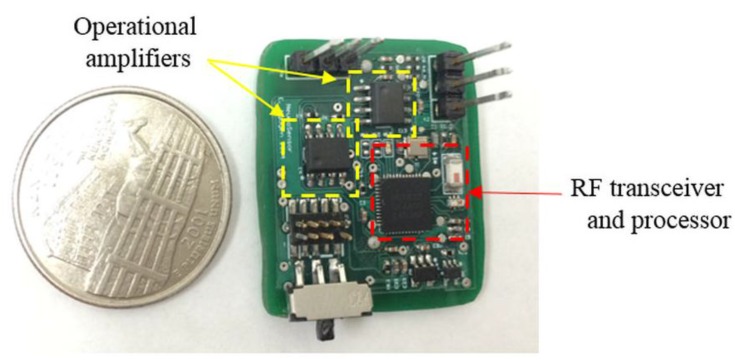
Photo of the neurotransmitter sensor recording device with a dimension of 25 × 27 × 6 mm^3^. The module included multi-stage amplifiers and a system-on-chip (SoC) processor with an integrated radio-frequency (RF) transceiver.

A photo of the neurotransmitter sensor recording module alongside a U.S. quarter for size comparison shown in Figure 2. The module prototype had dimensions of 25 × 27 × 6 mm^3^. The module can be made smaller by using a thin printed-circuit board and smaller surface mount component footprints. Power consumption at the rated voltage of 2.5 V was 42 mW. The RF transceiver accounted for 62% of the total power consumption to maintain connectivity with the base station at a distance of up to 100 ft. The total amount of power required to operate the amplifier module including the buffer circuit, differential and transimpedance amplifiers was 26%. The processor and other miscellaneous components drained the remaining 7% of total power.

### 2.3. Wireless Power Transmission (WPT)

**Figure 3:**
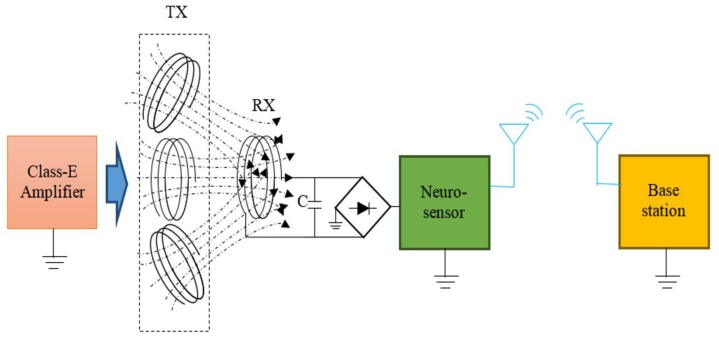
Block diagram of the wireless power harvester for the wearable neurotransmitter sensor module.

The neurotransmitter sensor recording system was designed to be powered by a WPT system. The block diagram of the WPT system is shown in Figure 3. A class-E amplifier was driven by an external power supply to convert a DC source into RF energy, which was transmitted through an array of transmitter antennas. Figure 3 illustrates how multi-transmitter antennas (TXs) were used to guarantee sufficient energy transfer to the receiver antenna (RX) at any location in the animal confinement space. The RX inductively coupled with the TX at the same resonant frequency to harvest RF energy. The harvested energy was then converted into DC power using a rectifier and supplied the neurotransmitter sensor module. A super-capacitor C was used to store the energy temporarily. Since the neurotransmitter sensor module was worn by a freely-moving animal, the distance between the TX and RX was changed constantly and randomly during experiments. Harvested energy thus was affected by lateral and angular misalignments of the antennas.

### 2.4. Experiment Setup

**Figure 4:**
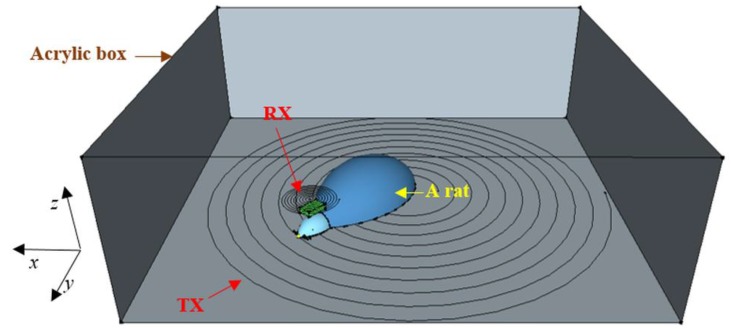
Illustration of the experiment setup with a freely-moving rat wearing the neurotransmitter sensor module inside an acrylic box with a dimension of 40 × 40 × 15 cm^3^.

The experiment setup was designed to measure L-Glu signals in a freely-moving rat that would be kept in an acrylic box with dimensions of 40 cm × 40 cm × 15 cm, as depicted in Figure 4. Two-month old Sprague-Dawley rats were chosen as the animal models for this type of experiments. For simulation configurations, the movement of the rat was limited within a virtual box with a space of 40 cm × 40 cm and a 10-cm height. Misalignments in position and angle between the TX and RX antennas were critical in the study. The WPT was required to provide sufficient energy in the case of greatest misalignment. In Figure 4, the lateral misalignment was illustrated by moving the RX antenna in the *x-*, *y*- and *z*-direction while angular misalignment was introduced by rotating the RX antenna along the *x*-axis from 0° to 90°. Harvested energy was measured in each case with a resistive load of 745 Ω. The load was chosen based on the input impedance of the neurotransmitter sensor module in its sleep mode.

A TX antenna with an outer radius of 20 cm was placed underneath the acrylic box. It had a spiral shape with the parameters described in Table 1. A recent study showed that the spiral-shaped TX antenna was able to provide a better beam size and had improved power efficiency [45,46,47]. Although Figure 4 illustrates a spiral-shaped RX coil, the final RX antenna was a 60-turn radial coil with a 1.6-cm radius and weight of around 13 grams as it had a smaller size to fit on the rat’s head. The TX antenna was made of Litz wire while the RX antenna used AWG 24 wire. The operating frequency is 1.3 MHz [45,48,49].

**Table 1:** Antenna information.

Parameters	Transmitter	Receiver
Inner radius (cm)	7	1.6
Outer radius (cm)	20	–
Turn number	20	60
Turn spacing (cm)	0.65	–
Inductance (µH)	142.3	239.3
Resistance (Ω)	20.5	212.2
Quality factor	56	8.3

### 2.5. In Vitro Experiment Protocol

As a preliminary step to demonstrate that the wirelessly powered module could work properly with the wireless L-Glutamate sensor implant module, we utilized a benchtop setup mimicking animal models to create controlled scenarios. Using animals directly, we would face: (1) the trouble of controlling the position and duration of stay of the rat in a certain location to recorded harvested power which could alter the animal’s behaviors; and (2) the challenge of creating calibrated electrical signals in response to L-Glu concentration variations since the L-Glu in brain toward certain environmental stimuli do not follow a deterministic function.

The experiments were then conducted in a controlled scenario with a small beaker containing 40 mL of phosphate buffered saline solution (Sigma-Aldrich). The temperature of the beaker was kept constant at 37 °C using a water bath. L-Glu (Fluka Analytical) was gradually added in steps to raise the total concentration of the L-Glu in the beaker by 50 µM every three minutes. A magnetic rod continuously stirred the solution to maintain homogeneity. An L-Glu sensor was placed inside the beaker to monitor the concentration of the L-Glu. The neurotransmitter sensor module was connected to the L-Glu sensor with short copper wires. The room temperature was controlled at 25 °C to eliminate any effect of thermal noise during recording. The wireless recording module integrated with the RX coil was placed inside the acrylic box at *x* = 0 cm and *y* = 0 cm at a height of *z* = 10 cm. One TX antenna configuration was used in this experiment. The module was then powered by the WPT system at an input voltage of 10 V. Wireless communication was established with a computer at the base station. The neurotransmitter sensor module operated and transferred data to the base station when it moved with a step of 4 cm every 5 s within its working area as determined in the following section. Electrical current measured at the input of the transmitter amplifier circuit varied within a range of 0.51–0.53 A due to mutual coupling variations when the neurotransmitter sensor module moved around the box.

## 3. Results and Discussion

### 3.1. One-Transmitter Antenna Configuration

#### 3.1.1. Simulation

Reliability of continuous and sufficient power is required in our design. However, harvested energy of a WPT system is sensitive to the relative distance and orientation between the TX and RX antennas. It is important to investigate the magnetic field distribution generated by the TX antenna to understand energy available to the RX antenna at any location. Simulations were conducted in MATLAB software with the assumption that the electrical current was uniformly distributed throughout the TX coil [45]. The magnetic field intensity generated by the *i*th loop of the spiral TX coil includes tangential *H_pi_* and normal *H_zi_* field components, which are determined as [45,48]:

(5) $$H_{pi}=\frac{1}{2\pi }I\frac{z}{p{\left[{\left(R_{i}+p\right)}^{2}+z^{2}\right]}^{\frac{1}{2}}}*\left[-K_{i}\left(k\right)+\frac{R_{i}^{2}+p^{2}+z^{2}}{{\left(R_{i}-p\right)}^{2}+z^{2}}E_{i}\left(k\right)\right]$$

(6) $$H_{zi}=\frac{1}{2\pi }I\frac{1}{{\left[{\left(R_{i}+p\right)}^{2}+z^{2}\right]}^{\frac{1}{2}}}*\left[K_{i}\left(k\right)+\frac{R_{i}^{2}-p^{2}-z^{2}}{{\left(R_{i}-p\right)}^{2}+z^{2}}E_{i}\left(k\right)\right]$$

where *I* is the current flowing in the loop antenna, *R_i_* is the coil radius of the *i*th loop, and *p* and *z* are the displacements in tangential and normal directions, respectively. *K_i_*(*k*) and *E_i_*(*k*) in Equations (5) and (6), representing the elliptical integrals of first and second kinds to the modulus *k* of the *i*th loop [45], also depend on the tangential and normal displacements.

**Figure 5:**
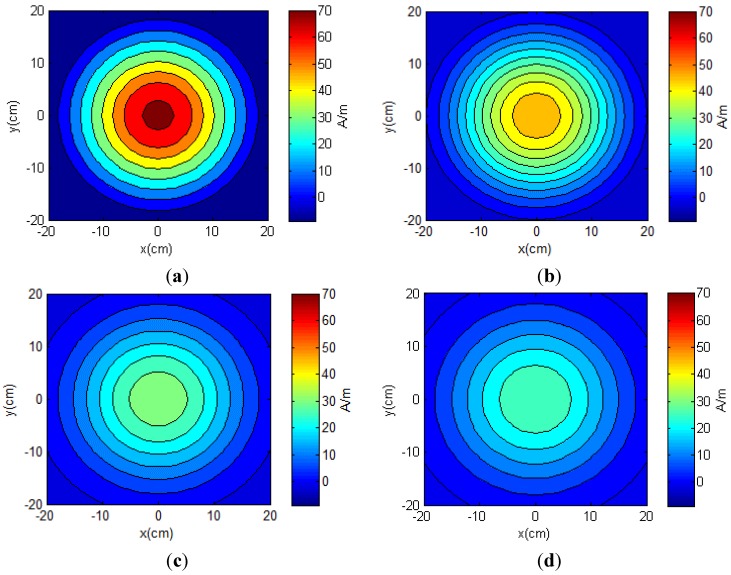
Radiation patterns of the normal field components *H_z_* generated by the transmitter (TX) spiral antenna at different distances *z* of (**a**) 4, (**b**) 7, (**c**) 10 and (**d**) 11 cm.

Radiation pattern of the TX antenna was analyzed with an electrical current of 1 A flowing in each loop. When the RX antenna was much smaller than the TX antenna and was in parallel with it, only the normal field component *H_z_* contributed to the energy harvested [45,48]. Figure 5 illustrates the magnetic field distributions of the normal field components *H_z_* in the cross-section planes at different distances *z* of 4, 7, 10 and 11 cm from the TX antenna. The normal field component of each plane *z* was strongest at the center (*x* = *y* = 0 cm). The normal field *H_z_* decayed with increase in the distance *z*. For example, the maximum values of the magnetic fields were 71.27, 49.02, 33.71 and 29.87 A/m with the distances *z* = 4, 7, 10 and 11 cm, respectively. Since the magnetic fields directly affected the induced voltage at the RX coil, the harvested energy was expected to have a similar pattern.

#### 3.1.2. Lateral Misalignment

**Figure 6:**
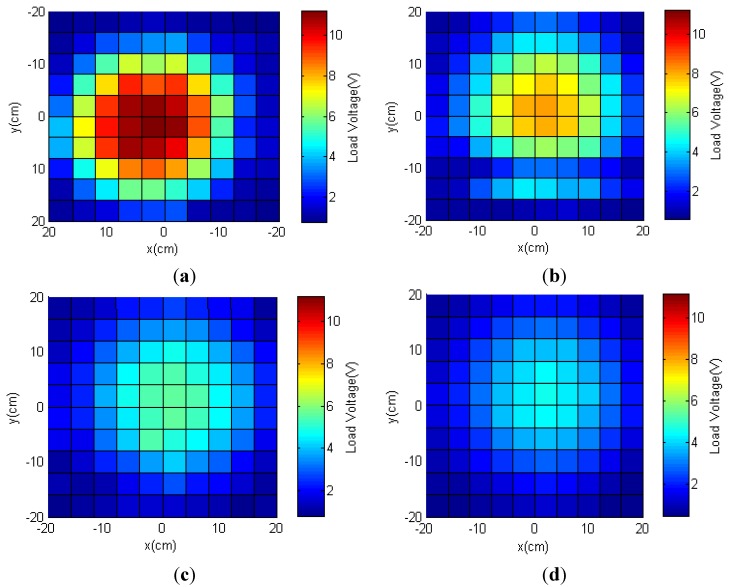
Measured load voltages at different distances *z* of (**a**) 4; (**b**) 7; (**c**) 10 and (**d**) 11 cm when a load of 745 Ω was connected to the receiver side.

Experiments were designed to measure harvested power on the receiver. A resistive load of 745 Ω matching the input impedance of the neurotransmitter sensor module in the sleep mode was connected to the receiver module. The RX antenna was tuned at 1.3 MHz and kept parallel to the TX antenna. Load voltages were recorded while the receiver moved two-dimensionally along *x*- and *y*-directions every 4 cm. Figure 6 shows the load voltages mapped at different distances *z* of 4, 7, 10 and 11 cm. The class-E amplifier at the transmitter side was operated by a DC power supply to maintain a voltage of 10 V and a current of around 0.5 A. The current was monitored and recorded as it fluctuated a little due to mutual coupling variation between the antennas.

Although the processor of the neurotransmitter sensor module could operate at a voltage as low as 1.6 V, it required a higher voltage level to be activated from the sleep mode. This threshold voltage was estimated as 2.0 V. In our application, the total area with load voltages equal or higher than 2.0 V determined the working area where the module could be activated and work properly. Results of peak powers at the center in Figure 6 agreed with the simulation results in Figure 5.

The effective coverage was the ratio of the working area to the total area inside the acrylic box. Measurements showed effective coverage at distances *z* of 4, 7, 10 and 11 cm were 54.5, 56.2, 55.3 and 38.02%, respectively. Effective coverage was maintained at around 55% when the distance *z* was less than 10 cm. In other words, the module still operated properly when the RX antenna moved around within a circle with a diameter that was 74% of the TX antenna diameter. When the RX antenna was moved to a distance *z* = 11 cm, we observed a drop in the effective coverage. At this distance, the fields became weak; however, the neurotransmitter sensor module could still operate properly within 61% of the circular area of the TX antenna.

#### 3.1.3. Angular Misalignment

**Figure 7:**
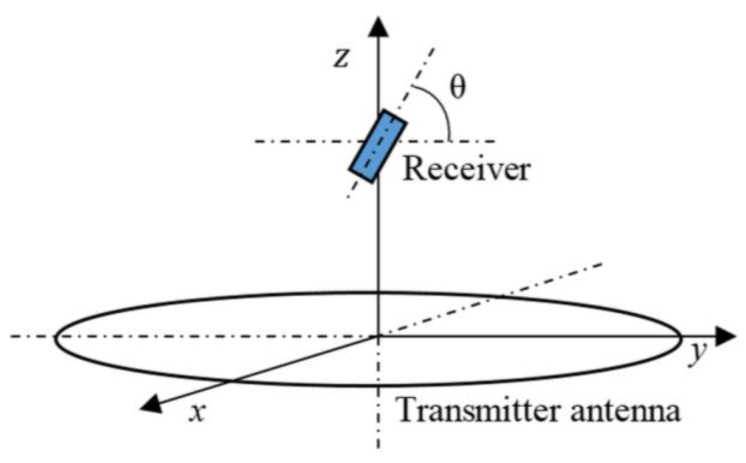
Angular misalignment θ between the TX and receiver (RX) antennas.

In the practical scenario, angular misalignment between antennas occurred when the freely-moving rat carrying the wireless module moved its head. Figure 7 illustrates the angular misalignment θ between the TX and RX antennas. Since the spiral TX and radial RX antennas were symmetric, the angular misalignment θ was made by rotating the RX antenna along the *x*-axis to form an angle θ with the *y-*axis. The experiments were repeated by moving the receiver along *x*- and *y*-directions every 4 cm with the same angular misalignment θ. The load voltages were repeatedly measured at different distances *z* between the TX and RX antennas.

**Figure 8:**
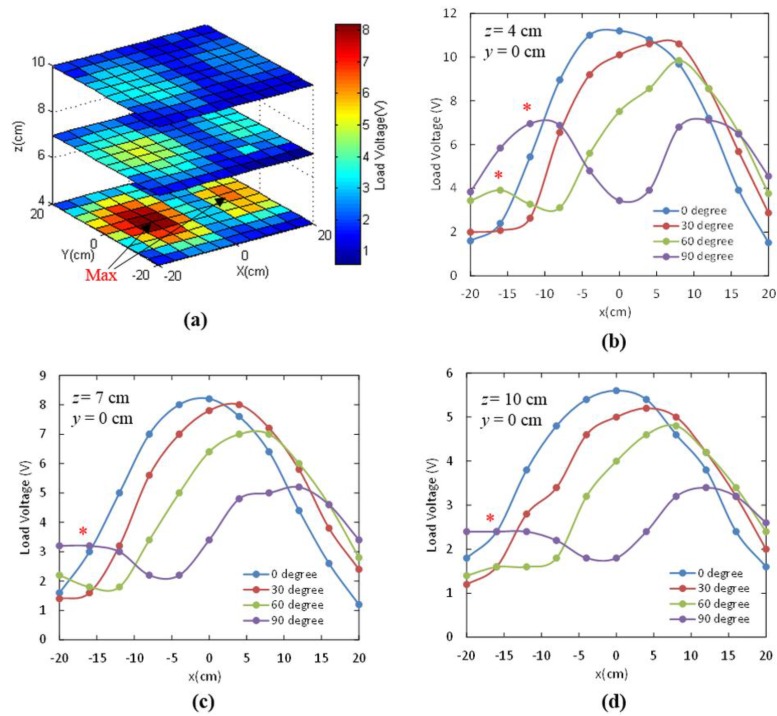
Measured load voltages at different distances *z* = 4, 7, 10 cm when a load of 745 Ω was connected to the receiver side. (**a**) The angle θ between the TX and RX antennas was 90°. Measured load voltages at the planes of *y* *=* 0 cm and (**b**) *z* *=* 4 cm; (**c**) *z =* 7 cm; and (**d**) *z* *=* 10 cm; with different angular misalignment θ *=* 0°, 30°, 60° and 90°.

Figure 8a shows the load voltages measured at different planes with *z* = 4, 7 and 10 cm when the angular misalignment θ = 90°. The maximum load voltages were not achieved at the center of the TX antenna but were distributed towards the left and right sides. This agrees with the investigation in [45] because the RX antenna harvests the tangential component instead of the normal component of the fields. The total tangential field component reached maximum at a distance off the center. Figure 8b shows the measured load voltages at different angular misalignments θ when the module moved in the *x-*direction, while maintaining *z* *=* 4 cm and *y* = 0 cm. The locations of the maximum load voltages shifted toward the right when the angular misalignment θ increased from 0° to 90°. The maximum voltage that could be harvested also decreased as the location shifted. Specifically, the maximum load voltages were located at *x* *=* 0, 4, 8 and ±12 cm for the angular misalignment θ = 0°, 30°, 60° and 90°, respectively. We also observed a second peak load voltage, marked as (*), which became obvious with increase in angular misalignment θ, particularly at 60° and 90°. Similar phenomenon was also observed at the distances *z* of 7 and 10 cm in Figure 8c,d, although less pronounced. Higher load voltages toward the side were due to the contribution of the total tangential field component. The *H_p_* component only induced small energy in the RX antenna when both antennas were parallel [45]. However, when the receiver antenna was rotated, the contribution of the tangential *H_p_* component became more significant than the normal *H_z_* component, which otherwise decreased due to misalignment.

The effective coverage with angular misalignment is shown in Table 2. The effective coverage increased when the angular misalignment θ increased at short distance *z* *=* 4 cm. Both *H_p_* and *H_z_* field components were large at such a close distance; therefore, the rotation of antennas coupled more energy. An effective coverage of 78.1% at θ = 90° was achieved. Additionally, the strongly coupled fields occurred towards the edges of the TX antenna instead of being at the center. When the distance *z* increased, the two components decreased significantly due to field decay and divergence. However, the effective coverage in such cases did not change dramatically and only varied within 46%–55%.

**Table 2:** Effective coverage.

Angular Misalignment θ	*z =* 4 cm	*z =* 7 cm	*z =* 10 cm
0°	54.5%	56.2%	55.3%
30°	63.6%	54.1%	54.1%
60°	73.9%	52.1%	45.8%
90°	78.1%	63.6%	46.3%

### 3.2. Two-Transmitter Antenna Configuration

**Figure 9:**
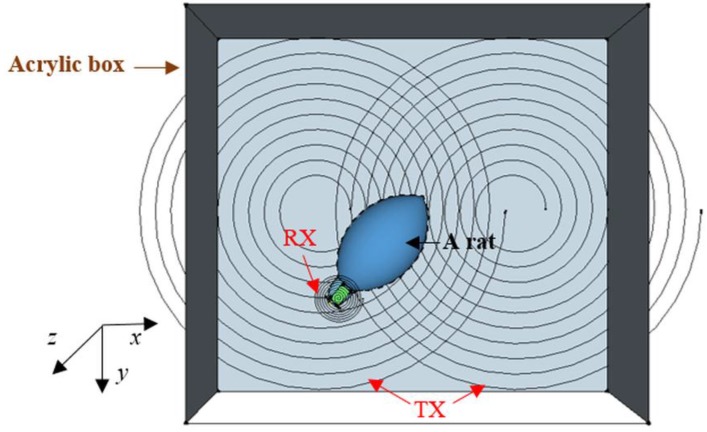
Two identical TX antennas were arranged 20 cm apart driven by the same power supply and identical amplifier circuitry.

A multi-TX antenna configuration was proposed to enhance effective coverage of the WPT system in the acrylic box for animal experiments, especially at the edges of the confinement box. The top-view of the two-antenna configuration is illustrated in Figure 9. The parameters of the TX antennas are the same as the ones listed in Table 1. The distance between the centers of two antennas was 20 cm. They were connected to the same current source.

#### 3.2.1. Simulations

**Figure 10:**
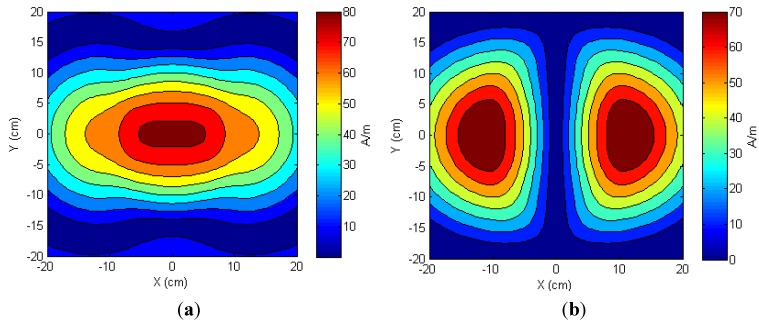
Simulation of the normal component of magnetic fields at a distance *z* = 4 cm from the two TX antennas when two antennas were (**a**) in-phase and (**b**) out-of-phase.

Simulations of the magnetic field distribution generated by two identical antennas using MATLAB are shown in Figure 10. An electric current of 1 A was set to flow through the antennas. The normal field component of the magnetic field was plotted at a distance *z* *=* 4 cm from the TX antennas. When the two TX antennas were in-phase, the magnetic fields were not only focused at the center of each antenna, but also overlapped the area between them (Figure 10a). When the currents were out of phase, the magnetic fields from these antennas canceled at the middle, as shown in Figure 10b. Intuitively, the in-phase TX antennas should provide a higher effective coverage and higher maximum magnetic field intensity.

#### 3.2.2. Measured Load Voltage

In experiments, the two identical TX antennas were tuned at 1.3 MHz and driven by the same power supply and identical amplifier circuitry. The load voltages were measured with a resistive load of 745 Ω connected to the rectifier of the receiver. The power supply maintained a voltage of 10 V and a current of 0.33 A for these antennas.

**Figure 11:**
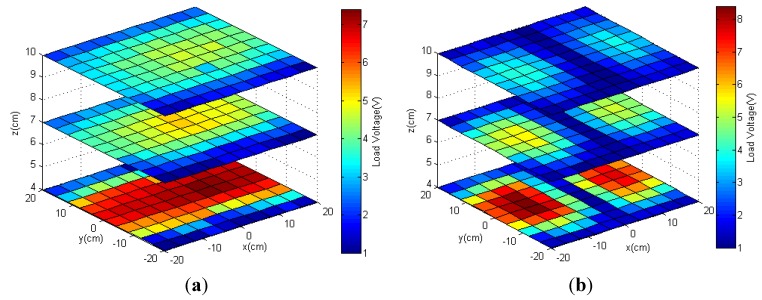
Measured load voltages at different planes at *z* = 4, 7 and 10 cm when using two TX antennas were (**a**) in-phase and (**b**) out-of-phase.

Figure 11 shows the measurement results, which agreed with the simulations. Significant increases in effective coverage with in-phase TX antennas were measured as 79.3%, 77.6% and 82.6% with distances *z* of 4, 7 and 10 cm, respectively. Importantly, the two-TX antenna configuration allowed the device to be powered at edges of the acrylic box, specifically at *x* *=* *±*20 cm, as shown in Figure 11a. Although the out-of-phase TX antennas induced a higher maximum load voltage (for example, 8.4 V at *z* *=* 4 cm), it formed a low-power area between these two antennas, as shown in Figure 11b. Since the magnetic fields were canceled in this area, the load voltage was not enough to activate the system.

### 3.3. Multi-Transmitter Antenna Configuration

**Figure 12:**
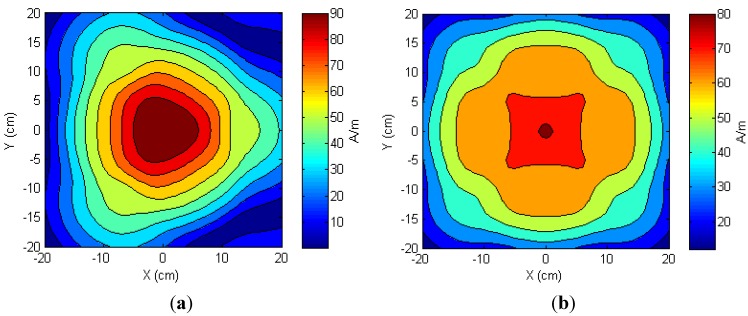
Simulation of the normal component of magnetic fields at a distance *z* = 4 cm from (**a**) three TX antennas; and (**b**) four TX antennas.

In the previous sections, we showed a close agreement between the measured load voltages and simulation results of the one- and two-TX antenna configurations. Although the simulation results did not directly correlate to a specific amount of harvested energy at the receiver side, they could be used to predict the working area and the pattern of the harvested power. The simulation was utilized to analyze the magnetic field distributions of multi-TX antenna configurations. We also assumed that the RX antenna was kept in parallel to and was much smaller than the TX antenna. Thus, we only considered the normal component of the magnetic field intensity. Figure 12a,b illustrate the normal *H_z_* component at the distance *z* = 4 cm obtained from three- and four-TX antenna configurations, respectively. These antennas were arranged symmetrically to the center (*x* = *y* = 0 cm) so that the distance between them was 20 cm. An electrical current of 1 A was applied to each TX antenna.

For comparison purpose, we defined a coverage percentage for the magnetic field intensity *H_z_* = κ, a constant number, as the ratio of the area where the normal field component is equal to or higher than κ to the total area of the acrylic box. Table 3 shows an increasing trend in the coverage percentage with higher numbers of the TX antennas. In particularly, the percentages of area having the normal *H_z_* field component equal to or higher than 30 A/m were 28.14, 48.70, 65.26 and 88.10%. These percentages corresponded to the one-, two-, three- and four-TX antennas, respectively. The four-TX antenna configuration also guaranteed that the entire area (100% coverage) has *H_z_* equal to or greater than 10 A/m, which should induce sufficient energy for the neurotransmitter sensor module to work properly. Simulation results confirmed that higher coverage could be achieved by placing more TX antennas in appropriate locations.

The multi-TX antenna configuration could help to improve effective coverage, especially at the edges of the acrylic box; however, phase matching among antennas became a critical issue. Increasing the number of the TX antennas required a complex tuning system and some efforts to match the phases of individual antennas. As more coils were involved, the mutual coupling among coils play a distinct role and the combination of self-inductance and mutual inductance made the tuning for resonance difficult. Another approach to improve the effective coverage is to increase the physical size of the TX antenna. In such a case, the total size of the system would be enlarged and it may become a concern for experiments.

**Table 3:** Coverage percentage.

Number of TX Antenna	*H_z_* ≥ 30 A/m	*H_z_* ≥ 20 A/m	*H_z_* ≥ 15 A/m	*H_z_* ≥ 10 A/m
One	28.14%	37.66%	43.13%	48.84%
Two	48.70%	58.60%	65.73%	76.80%
Three	65.26%	76.26%	83.46%	89.59%
Four	88.10%	98.97%	99.29%	100%

In our designs for animal experiments, the neurotransmitter sensor module has a 4-F super-capacitor to temporarily store energy. It could operate for 10 min in the full operation mode or 24 h in the sleep mode without getting the wireless power [50]. Since it was likely that animals would move around during a 24-h period, it was not a concern to have some areas with low power supply. With this scenario as a condition, the receiver was programmed to allow triggering the TX antenna to turn on or off to keep the super-capacitor within a desired voltage range. Implementation of such a protocol enabled power saving at the TX and reduced average Specific Absorption Rate (SAR) exposure, by which excessive RF energy might induce localized heating in the rat’s tissues.

### 3.4. *In Vitro* Measurement of the L-Glu Sensors

**Figure 13:**
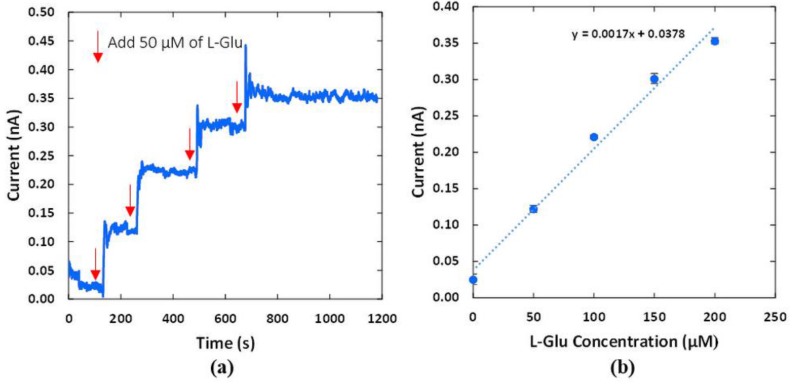
(**a**) Electrical current response of the L-Glu sensor which were wirelessly recorded with the neurotransmitter sensor module powered by the WPT system; (**b**) Calibration curve shows a sensitivity of 1.7 pA/µM with standard deviation (SD) less than 6.7 pA.

Electrical current responses of the L-Glu sensor corresponded to the simulated changes in the beaker. The integrated potentiostat converted the electrical current signals into voltages and the microprocessor transmitted the voltage signals to the base station [23]. The voltage values were updated at a rate of 10 samples/s and transmitted to the base station via wireless communication. These raw data was converted back to electrical current signals from the neurotransmitter sensor module with a preset gain of 10^9^ V/A. Figure 13a shows current sensing signals corresponding to the changes in concentration of L-Glu inside the beaker. The baseline response of the module was at 22.4 pA. There was a transient period at the onset of the experiment. Electrical current response decreased from 59 pA to the baseline after 2 min. This was due to hydration and stabilization on the surface of the L-Glu electrodes.

Figure 13a illustrates distinguishable current response when the L-Glu was added. Delay times of less than 30 s and signal overshoots were due to both the mixing of solution until equilibrium was attained and sensor responses. The current remained at 350 pA for 10 min when concentration of L-Glu was fixed at 200 µM. This shows the stability of the sensor and the WPT system. The sensitivity of the L-Glu recording module was calculated to be 1.7 pA/µM, as shown in Figure 13b. Standard deviation (SD) of the measurement was less than 6.7 pA. Noises were partially caused by the magnetic stirrer, which would not be present in animal experiments. The experiment demonstrated that the L-Glu sensor worked as expected with the wireless power transfer system for use in the acrylic box.

## 4. Conclusions

We have designed and demonstrated a wireless power transfer system for powering a wearable and wireless neurotransmitter sensor recording system. Simulation for electromagnetic field distribution and experimentally measured load voltages at various distances with different antenna configurations were in close agreement. A multi-antenna system as power transmitter provided a better effective coverage, especially at the edges inside the animal cage. The low-power neurotransmitter sensor recording module dedicated for L-Glu sensing was successfully powered and operated by the wireless power transfer system. The study demonstrated that the module can be autonomously operated without carrying battery packs on small animals in long-term experiments.
